# Slow-Absorbing Modified Starch before and during Prolonged Cycling Increases Fat Oxidation and Gastrointestinal Distress without Changing Performance

**DOI:** 10.3390/nu8070392

**Published:** 2016-06-25

**Authors:** Daniel A. Baur, Fernanda de C. S. Vargas, Christopher W. Bach, Jordan A. Garvey, Michael J. Ormsbee

**Affiliations:** 1 Institute of Sport Sciences and Medicine, Department of Nutrition, Food, and Exercise Sciences, Florida State University, Tallahassee, FL 32306, USA; dab13b@my.fsu.edu (D.A.B.); fd14c@my.fsu.edu (F.d.C.S.V.); cwb12b@my.fsu.edu (C.W.B.); jag12h@my.fsu.edu (J.A.G.); 2 Department of Biokinetics, Exercise and Leisure Sciences, University of KwaZulu-Natal, Durban 4000, South Africa

**Keywords:** glycemic index, gastrointestinal distress, blood glucose, ergogenic aids, carbohydrate

## Abstract

While prior research reported altered fuel utilization stemming from pre-exercise modified starch ingestion, the practical value of this starch for endurance athletes who consume carbohydrates both before and during exercise is yet to be examined. The purpose of this study was to determine the effects of ingesting a hydrothermally-modified starch supplement (HMS) before and during cycling on performance, metabolism, and gastrointestinal comfort. In a crossover design, 10 male cyclists underwent three nutritional interventions: (1) a commercially available sucrose/glucose supplement (G) 30 min before (60 g carbohydrate) and every 15 min during exercise (60 g∙h^−1^); (2) HMS consumed at the same time points before and during exercise in isocaloric amounts to G (Iso HMS); and (3) HMS 30 min before (60 g carbohydrate) and every 60 min during exercise (30 g·h^−1^; Low HMS). The exercise protocol (~3 h) consisted of 1 h at 50% W_max_, 8 × 2-min intervals at 80% W_max_, and 10 maximal sprints. There were no differences in sprint performance with Iso HMS vs. G, while both G and Iso HMS likely resulted in small performance enhancements (5.0%; 90% confidence interval = ±5.3% and 4.4%; ±3.2%, respectively) relative to Low HMS. Iso HMS and Low HMS enhanced fat oxidation (31.6%; ±20.1%; very likely (Iso); 20.9%; ±16.1%; likely (Low), and reduced carbohydrate oxidation (−19.2%; ±7.6%; most likely; −22.1%; ±12.9%; very likely) during exercise relative to G. However, nausea was increased during repeated sprints with ingestion of Iso HMS (17 scale units; ±18; likely) and Low HMS (18; ±14; likely) vs. G. Covariate analysis revealed that gastrointestinal distress was associated with reductions in performance with Low HMS vs. G (likely), but this relationship was unclear with Iso HMS vs. G. In conclusion, pre- and during-exercise ingestion of HMS increases fat oxidation relative to G. However, changes do not translate to performance improvements, possibly owing to HMS-associated increases in gastrointestinal distress, which is not attenuated by reducing the intake rate of HMS during exercise.

## 1. Introduction

Carbohydrate is a well-documented ergogenic aid for endurance performance, and benefits are dose-responsive within intestinal absorption capacity limits (~90 g∙h^−1^) [1]. As such, current recommendations suggest athletes consume large amounts of carbohydrate before and during prolonged (≥120 min) exercise to optimize performance [2]. Notably, studies have found that endurance athletes generally comply with these recommendations [3,4]. 

Recent research has highlighted the importance of carbohydrate type on metabolic and performance outcomes. For instance, composite solutions containing glucose and fructose (1 to 0.8–1.0 ratio) ingested during exercise seem to enhance performance relative to glucose/maltodextrin-only solutions [5,6]. This effect is likely due to faster carbohydrate absorption and oxidation with glucose/fructose mediated by non-competitive intestinal transport [7]. Interestingly, there is also evidence that reducing the rate of carbohydrate absorption with slow-digesting carbohydrate benefits exercise metabolism and performance. For example, studies have reported enhanced endurance capacity (70% VO_2max_ to exhaustion) or time trial performance (pre-loaded 16-km run) following a pre-exercise meal composed primarily of slow-absorbing and/or low glycemic index carbohydrates [8,9]. This ergogenic effect may be the result of more efficient substrate utilization patterns. Indeed, reported performance improvements are often associated with enhanced exercise fat oxidation, possibly resulting from attenuated blood glucose and insulin responses to feeding [8,9]. Benefits may also be partially explained by a prolonged glucose release from the small intestine maintaining euglycemia during exercise and consequent attenuation of central fatigue [10]. Of interest, this extended energy release into the bloodstream combined with enhanced fat oxidation may permit the intake of fewer overall carbohydrates during exercise. If true, this may be desirable for certain athletes, as carbohydrate intake rates, while associated with performance benefits, are also associated with gastrointestinal distress [3].

Nevertheless, the utility of slow-absorbing carbohydrates for endurance athletes is uncertain. Even if potentially required in lesser amounts, the carbohydrate demands of endurance exercise necessitate ingesting carbohydrate both before and during exercise to maximize performance [11]. Most slow-absorbing carbohydrates are in starch form, a semi-crystalline granular polymer typically found in whole foods like legumes, potatoes, and lentils [12]. Thus, because of the physical form of most starches, athletes are likely limited in their capacity to consume them during exercise due to logistical and palatability concerns. Moreover, any benefits conferred by pre-exercise slow-absorbing carbohydrate are substantially attenuated when traditional fast-absorbing carbohydrates are consumed during exercise [13]. As such, realizing the benefits of a pre-exercise slow-absorbing carbohydrate meal likely requires the continued intake of slow-absorbing carbohydrates during exercise. 

Importantly, the macromolecular structure of starch can be modified via various processing techniques to alter its solubility and rate of absorption. Recently, a slow-absorbing and water-soluble waxy maize starch-based exercise supplement was developed through hydrothermal modification. Roberts et al. [14] found that, relative to maltodextrin, ingestion of this modified starch 30 min prior to cycling resulted in very likely increased fat oxidation combined with increased plasma concentrations of free fatty acids (FFA) and glycerol. While endurance capacity in a 100% VO_2max_ time to exhaustion trial following 150 min of cycling (70% VO_2max_) was unchanged with pre-exercise modified starch, subjects in the study did not consume additional carbohydrate during exercise despite the lengthy nature of the exercise protocol. This may have attenuated any performance benefit stemming from early exercise metabolic alterations. Furthermore, this feeding strategy contrasts with current recommendations and current practice among athletes. As such, the purpose of this study was to investigate the impact of consuming a slow-absorbing modified starch supplement both before and during exercise relative to an isocaloric, fast-absorbing carbohydrate solution in trained male athletes. A secondary purpose was to determine whether the extended glucose release profile and associated metabolic effects of a slow-absorbing modified starch permits the ingestion of less total carbohydrate without impairing performance. 

## 2. Methods

### 2.1. Subjects

Ten trained male cyclists and triathletes (age = 26 ± 8 years, mass = 75.2 ± 9.2 kg, VO_2max_ = 59.4 ± 3.2 mL∙kg^−1^∙min^−1^, and peak power (W_max_ = 343.3 ± 37.7 W) participated in the study. All subjects had ≥2 years cycling experience and had cycled ≥ 3 day∙week^−1^ and ≥ 7 h∙week^−1^ for the preceding two months, while regularly competing in races. Prior to giving their oral and written informed consent, all subjects received information regarding the requirements of the study and potential risks. All procedures were approved by the Florida State University Institutional Review Board. 

### 2.2. Study Design

This was a double-blinded, randomized, counterbalanced, and crossover study. It consisted of baseline testing to determine VO_2max_ and W_max_, a familiarization trial, and three experimental trials. Each experimental trial was separated by seven days. For the duration of the study, subjects were asked to maintain consistent dietary and training habits. Prior to each experimental trial, exercise and diet were standardized. Specifically, two days prior to each experimental trial, subjects visited the laboratory and completed a standardized training ride (90 min at 50% W_max_). The day prior to each trial, subjects were asked to refrain from exercise. Additionally, subjects were asked to replicate their diets (2444 ± 609 kcals; 103 ± 23 g protein, 299 ± 123 g carbohydrate, 97 ± 33 g fat) the day prior to each trial. This was achieved by requiring subjects to complete a 24 h dietary log prior to the first trial. Following this trial, subjects were given a copy of their completed dietary log and asked to replicate it exactly for proceeding trials. Subjects were also asked to abstain from alcohol and caffeine for the 24 h preceding each trial. 

### 2.3. Baseline Testing and Familiarization

During the initial visit to the laboratory, subjects were assessed for VO_2max_ and W_max_. This consisted of a continuous graded exercise test to exhaustion on a cycle ergometer (Velotron, Racermate, Inc., Seattle, WA, USA). During a self-selected warm-up, a power output corresponding to a “moderately difficult intensity for a 1 h ride” was determined. Commencing at this intensity, power was increased by 25 W every 2 min until volitional exhaustion. VO_2max_ was assessed with a calibrated metabolic cart (TrueOne 2400, Parvo Medics, Inc., Sandy, UT, USA) and was classified as the highest average 20-s oxygen consumption (mL∙kg^−1^∙min^−1^) recorded. W_max_ was the wattage attained in the last completed stage plus the fraction completed of the stage at which exhaustion occurred. 

A familiarization trial was completed 2–3 days following baseline testing. The familiarization trial consisted of the entire exercise protocol (see below) without the recording of data. 

### 2.4. Experimental Beverages

The current study evaluated two commercially available sport supplements. Specifically, we investigated the impact of ingesting different amounts of a hydrothermally-modified waxy maize starch (HMS; UCAN^®^, The UCAN Co., Woodbridge, CT, USA) relative to a sucrose- and glucose-based control solution (G; Gatorade^®^, PepsiCo, Inc., Purchase, NY, USA). Treatments were as follows: (1) 10% G consumed 30 min before exercise and 7.5% G every ~15 min during exercise; (2) 10% HMS consumed 30 min before exercise and 7.5% HMS every ~15 min during exercise (Iso HMS); and (3) 10% HMS consumed 30 min before exercise and 15% HMS every 60 min (at 60 min and following sprint two of the performance test) during exercise (Low HMS). The dosing strategy for the Low HMS trial was chosen based on recommendations available on the company’s website. In order to blind subjects to the dosing strategy, a non-caloric placebo was also ingested in the Low HMS condition during exercise at time points that matched G and Iso HMS beverage ingestion times. All beverages were flavor and texture-matched by the addition of non-caloric additives (e.g., sucralose and guar gum). Pre-exercise beverages contained 600 mL of fluid while during-exercise beverages were 200 mL. As such, carbohydrate delivery rates for G and Iso HMS were 60 g before and 60 g∙h^−1^ during exercise. For Low HMS, 60 g carbohydrate was ingested before and 30 g∙h^−1^ during exercise. These carbohydrate delivery rates were chosen as they represent the uppermost (60 g·h^−1^) and lowermost (30 g∙h^−1^) amounts of the currently recommended range for during-exercise ingestion of carbohydrate from a single source [15]. Beverage osmolality was determined via the freezing point depression method (Model 3250 Osmometer, Advanced Instruments, Inc., Norwood, MA, USA). Osmolalities were 363, 278, 51, 37, 53, and 8 mOsm∙kg^−1^ for pre-exercise G, during-exercise G, pre-exercise Iso/Low HMS, during-exercise Iso HMS, during-exercise Low HMS, and placebo, respectively. 

### 2.5. Experimental Trials

**Figure 1:**
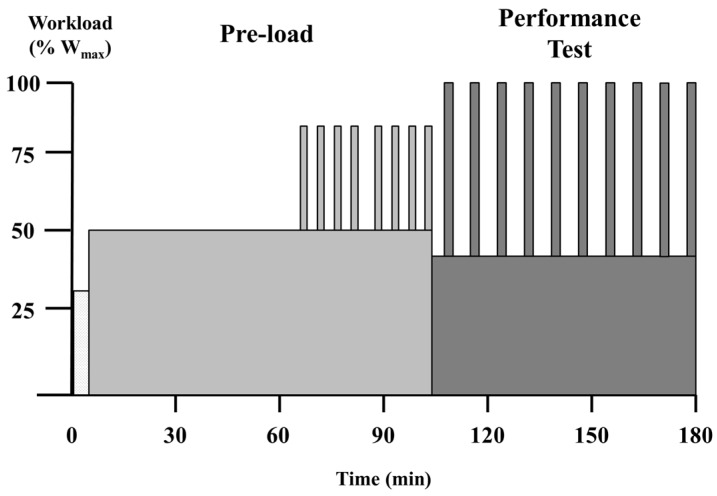
Exercise protocol. W_max_, peak cycling power.

Subjects reported to the laboratory at 0500–0700 h following an overnight fast (8–10 h). Arrival times were replicated for subsequent trials. Following 5 min of rest in the seated position, resting heart rate (Polar^®^ FTM4, Polar, Inc., Kempele, Finland) was assessed and a fingerprick blood sample was collected for immediate measurement of blood glucose and lactate (YSI 2300 Stat, YSI, Inc., Yellow Springs, OH, USA). Thereafter, a 5-min indirect calorimetry measurement was taken with the final 3 min being used in subsequent analysis. Subjects then received a pre-exercise treatment beverage, which they consumed within 3 min, and remained seated for 30 min. Blood and indirect calorimetry measurements were repeated 15 min and 30 min following ingestion. Next, subjects commenced exercise beginning with a 5-min warm-up at 30% W_max_. The exercise protocol is presented in Figure 1. It consisted of a 95-min pre-load after which subjects were allowed to stretch and use the restroom (3–5 min). This was followed by a repeated maximal sprint performance assessment, which has been previously described [6]. Specifically, the entire protocol consisted of the following: (1) 60 min at 50% W_max_; (2) two sets of 4 × 2-min intervals at 80% W_max_ with intervals and sets separated by 2 min and 5 min at 50% W_max,_ respectively; and (3) 10 maximal sprints assessed for mean power. For each sprint and recovery period of the performance test, subjects were required to complete a given amount of work based on their W_max_ (kilocalories = 0.125 × W_max_). For the sprints, subjects completed the prescribed work as quickly as possible (2–3 min). During recovery periods, the work was completed while subjects cycled at 40% W_max_ (5–6 min). Total exercise time was 183.0 ± 2.9 min.

Treatment beverages were consumed every 15 min during the pre-load portion and at the start and every second sprint during the performance assessment. Physiological measurements were as follows: (1) heart rate was measured every 15 min during the first 60 min of exercise and at the midpoint of each sprint and recovery segment of the performance test; (2) indirect calorimetry measurements were taken every 15 min for 5-min collection periods during the first 60 min of exercise; and (3) blood glucose and lactate were assessed every 15 min during the first 60 min of exercise, and following sprint 5 and sprint 10. 

All testing was completed in thermoneutral conditions (22 °C, 45%–50% humidity). Subjects were cooled by a pedestal fan on the medium setting in each trial for uniform cooling. During the performance testing portion of the exercise protocol, subjects received no verbal encouragement and were only permitted to see the amount of work completed. 

### 2.6. Perceptual Response Assessment

Gastrointestinal distress (nausea, abdominal cramp, and fullness) and perceived exertion (effort of cycling, tiredness, and leg strength) were assessed via a 100-mm Likert scale, as previously described [6,16]. Specifically, subjects rated the magnitude of these symptoms by placing a line in relation to specific descriptors including: nothing at all, extremely weak, very weak, weakor mild, moderate, strong, very strong, extremely strong, and absolute maximum. The height (mm) of the line marked by subjects was recorded for subsequent analysis. All measurements of line height were made via ruler by the same researcher. Perceptual responses were assessed every 15 min during the first 60 min of exercise, at the midpoint of the 5-min recovery period between 80% W_max_ intervals, and after the first and every third sprint of the performance test. 

### 2.7. Calculations

Total carbohydrate and fat oxidation at rest and during the first 60 min of exercise were calculated from indirect calorimetry measurements via stoichiochemical equations described elsewhere [17].

### 2.8. Statistics

Sample size was determined as that which provided sufficient power to detect the smallest worthwhile benefit to cycling performance given the expected typical error (CV) for mean sprint power and anticipated effect size (ES) [18]. Prior studies have reported CV of 1.1%–3.1% for mean sprint power [6,19,20]. To account for inter-laboratory differences, we chose to conservatively estimate a CV of 3.1% with an anticipated moderate 0.9 CV effect size (2.79%). Using 0.5% and 25% as the rates for Type I and Type II clinical errors, respectively, a sample size of 10 was determined. 

Probabilistic magnitude-based inferences were utilized to assess physiological and perceptual changes via a published spreadsheet [21]. The spreadsheet derives confidence intervals based on the unequal variances *t* statistic. All physiological data (i.e., performance and metabolic variables) were analyzed following log-transformation to account for any heteroscedasticity of error. Perceptual response raw data were analyzed without transformation. Uncertainty for all variables was expressed as 90% confidence intervals. Changes in performance were evaluated with the clinical version of magnitude-based inferences in which clear effects are classified as having >25% chance of benefit and <0.5% chance of harm. All other variables were assessed non-clinically; differences were deemed unclear if confidence intervals overlapped thresholds for both small positive and negative effects. ES was determined by standardizing all differences to the SD of the control, and small sample bias was accounted for by dividing the control SD by 1 − 3 (4v − 1), where v is equal to the degrees of freedom [18]. Threshold values for assessing performance were as follows: 0.3 (0.93%), 0.9 (2.79%), 1.6 (4.96%), 2.5 (7.75%), and 4.0 (12.4%) for small, moderate, large, very large, and extremely large, respectively [18]. Thresholds for small, moderate, large, very large, and extremely large changes in all non-performance variables were 0.2, 0.6, 1.2, 2.0, and 4.0, respectively, multiplied by the SD of the control condition (or the mean SD of the control for a given time period (e.g., the entire performance assessment)). Likelihoods for reaching the substantial change threshold were classified as follows: 5%–25%, unlikely; 25%–75%, possible; 75%–95%, likely; 95%–99%, very likely; and >99%, most likely. Log-transformed data is presented as back-transformed mean (CV). All other data is presented as the mean ± SD (or confidence intervals, where indicated). Differences are described as clear if the probability of a difference is likely or higher and non-trivial in size.

To examine the mechanistic impact of gastrointestinal distress on performance outcomes, correlation coefficient values were calculated using Microsoft Excel by plotting changes in performance against changes in gastrointestinal distress variables. Correlation coefficient confidence intervals were calculated via an additional published spreadsheet [22]. Correlation coefficient strength was qualified as follows: small 0.1, moderate 0.3, large 0.5, very large 0.7, and extremely large 1.0 [18]. Covariate analysis was utilized to assess the impact of changes in gastrointestinal distress on performance. Specifically, a linear model was utilized to assess the impact of individual symptoms of gastrointestinal distress on performance by adding the change in symptom values as a covariate in the primary published spreadsheet [21]. To evaluate the combined effect of multiple gastrointestinal distress symptoms, linear and positional coefficients from a polynomial model were calculated using the LINEST function in Microsoft Excel. An overall gastrointestinal distress covariate was then calculated as the sum of each coefficient multiplied by their respective symptom for each subject. The effect of the covariate was classified as the impact of adjusting performance effects to the mean value of the covariate. The effect, independent of the covariate, was determined by adjusting the impact of the covariate to zero.

## 3. Results

### 3.1. Performance

**Figure 2:**
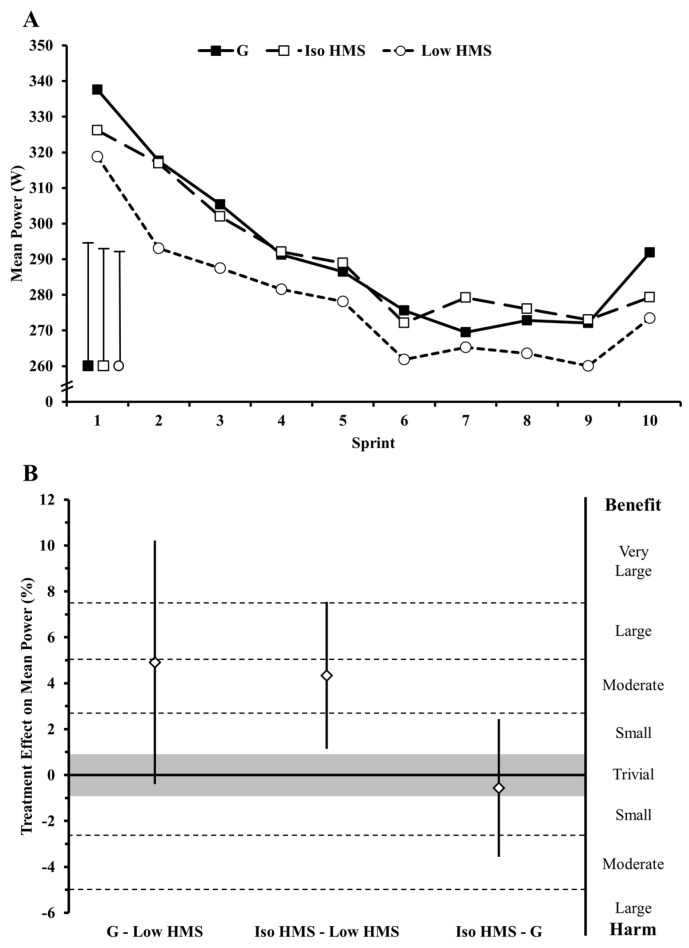
Effect of a hydrothermally-modified starch supplement on cycling performance. (**A**) Mean sprint power for each sprint of the performance test. Bars represent the mean standard deviation for all repeated sprints; and (**B**) mean effects (%) of treatment condition on mean sprint power. Bars represent the 90% confidence interval. G, a sucrose/glucose supplement; Iso HMS, an isocaloric dose (relative to G) of a hydrothermally-modified starch; Low HMS, low dose of a hydrothermally-modified starch.

Time course changes in sprint power and pairwise comparisons in mean sprint power are presented in Figure 2. Mean sprint power was 290.9 (10.8), 289.2 (10.7), and 276.0 (11.4) W for G, Iso HMS, and Low HMS, respectively. There were likely small increases in mean sprint power with G vs. Low HMS (ES = 0.46) and Iso HMS vs. Low HMS (ES = 0.40), respectively. Differences in mean sprint power with Iso HMS vs. G were likely trivial (ES = 0.05). 

### 3.2. Metabolic Parameters

Means and changes in VO_2_, total carbohydrate, and fat oxidation during rest and exercise are presented in Table 1. There were no clear differences in resting or exercise VO_2_. At rest and during exercise, Iso HMS (ES = 0.76 (rest) and 0.74 (exercise)) and Low HMS (ES = 0.73 and 0.63) enhanced fat oxidation relative to G. Additionally, Iso HMS (ES = 1.33 and 2.35) and Low HMS (ES = 1.77 and 2.20) reduced carbohydrate oxidation relative to G at rest and during exercise. Differences in substrate utilization with Iso HMS vs. Low HMS were unclear.

**Figure 3:**
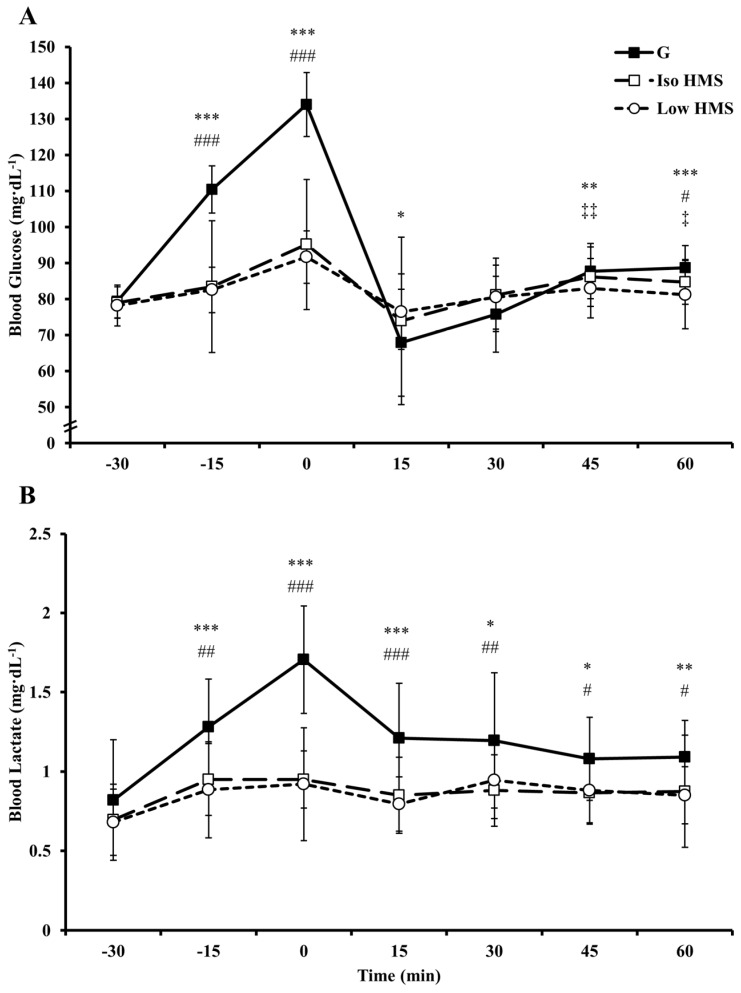
Time course changes in blood glucose and blood lactate. (**A**) Mean blood glucose values; and (**B**) mean blood lactate values. For (**A**,**B**), bars represent standard deviation. G, a sucrose/glucose supplement; Iso HMS, an isocaloric dose (relative to G) of a hydrothermally-modified starch; Low HMS, low dose of a hydrothermally-modified starch; *** denotes most likely different with G vs. Low HMS; ** denotes very likely different with G vs. Low HMS; * denotes likely different with G vs. Low HMS; ### denotes most likely different with G vs. Iso HMS; ## denotes very likely different with G vs. Iso HMS; # denotes likely different with G vs. Iso HMS; † denotes possibly different with G vs. Iso HMS; ‡‡ denotes very likely different with Iso HMS vs. Low HMS; ‡ denotes likely different with Iso HMS vs. Low HMS.

Time course blood glucose and lactate data are presented in Figure 3. For resting blood glucose, there were clear differences between HMS (Iso and Low) and G at −15 min (ES = 1.49 (Iso); 1.56 (Low)) and 0 min (ES = 1.64; 1.36). During steady-state exercise (0 min–60 min), blood glucose seemed to be higher with HMS vs. G at 15 min (ES = 0.36; 0.44), but was not clearly different at 30 min. Conversely, blood glucose was clearly higher with G vs. Low HMS at 45 min (ES = 0.53), and with G vs. HMS (Iso and Low) at 60 min (ES = 0.62; 1.14). There were no clear differences between HMS and G following sprint 5; however, blood glucose was very likely enhanced following sprint 10 with G vs. HMS (Iso and Low; ES = 0.77; 0.65). For Iso HMS vs. Low HMS, the only clear differences were at 45 min (ES = 0.36) and 60 min (ES = 0.51) where blood glucose was clearly elevated with Iso HMS. For lactate, HMS (Iso and Low) was clearly lower than G at rest (−15 min (ES = 1.13; 1.49), 0 min (ES = 2.56; 2.88)) and during steady state exercise (15 min (ES = 1.23; 1.41), 30 min (ES = 0.77; 0.57), 45 min (ES = 0.85; 0.77), and 60 min (ES = 1.04; 1.00)). The only clear difference during repeated sprints was a reduced blood lactate with Low HMS vs. G following sprint 10 (ES = −0.30). There were no differences in blood lactate levels between Iso HMS and Low HMS at any time point.

### 3.3. Heart Rate

There was a likely small and possibly small increase in mean heart rate during steady state exercise with G vs. Iso HMS (136 ± 7 vs. 133 ± 7; ES = 0.49) and G vs. Low HMS (136 ± 7 vs. 134 ± 6; ES = 0.25), respectively. There were no clear differences for mean heart rate during repeated sprints.

### 3.4. Perceptual Responses

**Figure 4:**
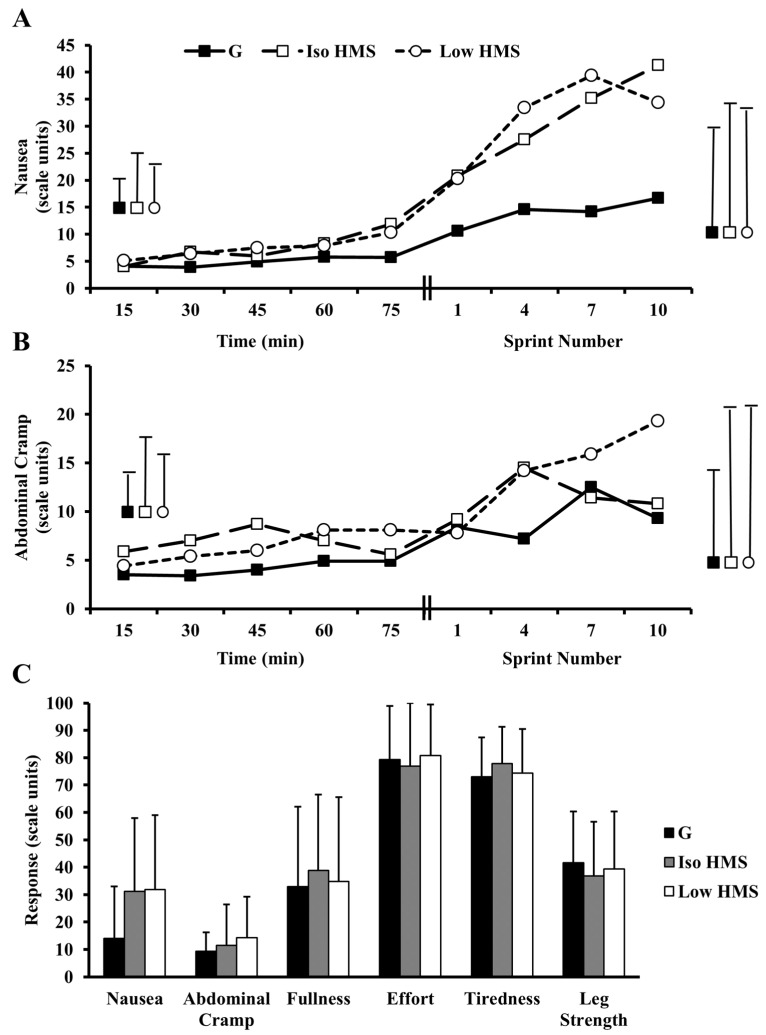
Changes in ratings of gastrointestinal distress and perceived exertion. (**A**) Ratings of nausea during exercise; (**B**) ratings of abdominal cramp during exercise. For (**A**,**B**), bars on the left represent mean standard deviation during the pre-load, and bars on the right represent mean standard deviation during the performance test; (**C**) Mean ratings of gastrointestinal distress and perceived exertion during the performance test. Specific changes are described in text. Mean nausea was likely increased with Iso and Low HMS vs. G during repeated sprints. Mean abdominal cramp was likely elevated with Low HMS vs. G during repeated sprints. Bars represent standard deviation. For effect magnitudes and inferences see text and Table 2. G, a sucrose/glucose supplement; Iso HMS, an isocaloric dose (relative to G) of a hydrothermally-modified starch; Low HMS, low dose of a hydrothermally-modified starch.

Time course changes in select gastrointestinal symptoms and differences in mean perceptual responses during repeated sprints are presented in Table 2 and Figure 4. There were clear differences for mean ratings of nausea during repeated sprints with HMS (Iso and Low) vs. G (31.2 ± 26.8 (Iso) and 31.9 ± 27.2 (Low) vs. 14.0 ± 18.9; ES = 0.83; 0.86). Additionally, mean ratings of abdominal cramp (14.3 ± 14.9 vs. 9.4 ± 6.9) were increased (ES = 0.65) with Low HMS vs. G during repeated sprints.

### 3.5. Gastrointestinal Distress-Mediated Effects on Performance

The influence of gastrointestinal distress on mean sprint performance is presented in Table 3. With Iso HMS vs. G, there were likely large correlations between mean sprint nausea (*r*= −0.51; ±0.45 (confidence interval)) and total gastrointestinal distress (nausea and abdominal cramp combined; *r* = −0.53; ±0.44) and performance. With Low HMS vs. G, there were very likely and most likely very large correlations for individual symptoms (nausea (*r* = −0.79; ±0.26) and abdominal cramp (*r* = −0.71; ±0.32)), and total gastrointestinal distress (r = −0.86; ±0.19) and changes in mean performance. Finally, there were very likely large correlations between nausea (*r* = −0.63; ±0.38) and total gastrointestinal distress (*r* = −0.65; ±0.37) and performance for Iso HMS vs. Low HMS. 

Adding gastrointestinal distress as a covariate revealed that changes in nausea and abdominal cramp mediated changes in performance. The influence of gastrointestinal distress increased the difference between G and HMS (Iso and Low) so that adjusting out the effects of gastrointestinal distress attenuated performance differences. Importantly, adjustment for gastrointestinal distress resulted in clear differences becoming unclear (G vs. Low HMS) or likely trivial impairments in performance becoming likely trivial enhancements (Iso HMS vs. G). The effects of individual symptoms were unclear or trivial; however, adjusting out either nausea or abdominal cramp altered inferences and/or effect magnitudes for performance.

## 4. Discussion

In prior research examining the effects of ingesting slow-absorbing carbohydrates on endurance performance, interventions have typically been confined to the pre-exercise window, likely as a consequence of carbohydrate physical form and palatability. This timing contradicts current nutritional guidelines and common practice among endurance athletes to ingest carbohydrate both before and during exercise. The present study examined the effects of ingesting a slow-absorbing HMS supplement both before and during exercise on exercise metabolism, gastrointestinal comfort, and high-intensity cycling performance. Primary findings were as follows: (1) fat oxidation was increased and carbohydrate oxidation decreased at rest and during exercise with HMS relative to G; (2) euglycemia was maintained with HMS relative to G; (3) performance was unchanged with ingestion of HMS relative to an isocaloric amount of G; (4) performance was impaired when the during-exercise ingestion rate of HMS was halved relative to G and Iso HMS; (5) incidences of gastrointestinal distress were increased with HMS ingestion; and (6) HMS-mediated increases in gastrointestinal distress seemed to be a major mechanistic determinant of changes in performance. 

Fat oxidation was enhanced and carbohydrate oxidation reduced with HMS ingestion relative to G in the current study. This finding is generally supported by studies examining pre-exercise slow-absorbing carbohydrate ingestion [14,23,24]. In the only other study to examine the effect of HMS ingestion on metabolic and performance outcomes, there was a very likely increase in fat oxidation combined with increases in plasma markers of lipolysis (i.e., glycerol and FFA) [14]. While this prior study did not report differences in total carbohydrate oxidation, our finding of reduced total carbohydrate oxidation is in line with a number of other studies examining pre-exercise intake of low glycemic index carbohydrate meals [23,24]. With during-exercise ingestion of slow-absorbing carbohydrates, metabolic findings are mixed. Specifically, increases in fat oxidation have been reported by some [16,25], but not others [26,27]. To our knowledge, this is the first study to examine the impact of a combined pre- and during-exercise slow-absorbing carbohydrate intervention. Importantly, a prior investigation revealed that ingestion of fast-absorbing carbohydrates (i.e., glucose) during exercise attenuates changes in substrate utilization induced by pre-exercise ingestion of a slow-absorbing carbohydrate meal [13]. Our data suggests that any pre-exercise-mediated alterations in substrate utilization induced by HMS are maintained (i.e., not attenuated) by continued during-exercise HMS intake. 

Differences in blood glucose responses and/or carbohydrate availability provide potential mechanisms for altered substrate utilization with HMS vs. G. With HMS, pre-exercise elevations in blood glucose were reduced ~20%–23% relative to G. Although not measured in the current study, this likely resulted in an attenuated elevation in insulin [8,9,14,28]. Further evidence comes from the substantially increased levels of blood lactate during exercise with G, which is likely attributable to enhanced blood glucose uptake and glycolysis mediated by insulin binding [29]. Importantly, insulin is potently antilipolytic providing a plausible, albeit speculative, mechanism for alterations in fat utilization [30]. Additionally, carbohydrate oxidation is heavily influenced by exogenous carbohydrate absorption rates [31]. With G, there were presumably substantially faster absorption rates relative to HMS due to non-competitive transport of glucose and fructose (products of sucrose) via separate intestinal transporters [7]. Moreover, digestion of HMS would be slower vs. G due to its increased complexity and/or extensive amylose/amylopectin branching which can impede amylase infiltration [12]. These factors likely enhanced carbohydrate delivery to skeletal muscle with G vs. HMS, thereby increasing carbohydrate oxidation at the expense of fat oxidation.

Despite substantial alterations in metabolism, performance was unchanged with Iso HMS relative to G. This finding is in agreement with Roberts et al. (2011) in which endurance capacity in a 100% VO_2max_ time to exhaustion bout following 150 min of submaximal cycling (70% VO_2max_) was unchanged with pre-exercise ingestion of HMS or maltodextrin (1 g∙kg^−1^) despite evidence for increased fat utilization with HMS. Additionally, a recent study by Oosthuyse et al. (2015) [16] found that, despite enhanced fat oxidation, cycling performance was impaired in a 16 km time trial following a 2 h pre-load (60% W_max_) with during-exercise isomaltulose (63 g∙h^−1^) compared to a maltodextrin/fructose composite. It is possible that enhancing fat oxidation with slow-absorbing carbohydrate (which would presumably be beneficial due to possible glycogen sparing [32]), simply does not translate to any meaningful changes in performance. Indeed, a number of studies have reported no change in time trial performance with a low glycemic index pre-exercise meal despite increased exercise fat oxidation [32,33]. Moreover, a recent study found that pharmacological abolishment of lipolysis via nicotinic acid infusion had no impact on half-marathon running performance suggesting that endurance performance may be primarily carbohydrate dependent [34]. 

It is also possible that any beneficial metabolic effects stemming from slow-absorbing carbohydrate intake are counterbalanced or overridden by non-metabolic mechanisms. For example, gastrointestinal distress was increased in the present study, and mechanistic analysis revealed this to be a negative, albeit unclear, mediator of performance with Iso HMS vs. G. In support, Oosthuyse et al. (2015) reported that during-cycling isomaltulose ingestion resulted in increased gastrointestinal distress coupled with impaired time trial performance. However, differences in performance in the current study with Iso HMS vs. G were trivial even after adjustment for gastrointestinal distress. As such, it is possible that the severity of symptoms was insufficient to alter performance, or that any negative impact of gastrointestinal distress may have been counterbalanced by metabolic benefits (e.g., enhanced fat oxidation). Another possibility is that the impact of gastrointestinal distress may be more apparent in time trial scenarios, which require persistent concentration and pacing, relative to repeated sprint protocols that are more unrestrained in nature [6]. This might help to explain clear performance impairments in the Oosthuyse et al. study, but unclear effects of gastrointestinal distress on performance with Iso HMS vs. G in the current study. However, this notion seems less likely considering the impact of gastrointestinal distress on performance with G vs. Low HMS (discussed below). Regardless, more research is clearly warranted to elucidate the precise impact of gastrointestinal distress on performance and how these effects are altered by metabolic factors. 

Perceptual response findings in the current study add further evidence to the notion that malabsorption is the primary pathophysiologic mechanism of carbohydrate-induced gastrointestinal distress during exercise. Indeed, while others have reported associations between beverage osmolality and gastrointestinal distress [35], symptoms of nausea in the present study were elevated despite very low solution osmolalities with Iso HMS and Low HMS vs. G (37–53 vs. 278–363 mOsm·kg^−1^). Similarly, others have reported clear differences in gastrointestinal comfort with during-exercise ingestion of slow- vs. fast-absorbing carbohydrates despite consuming solutions of the same approximate osmolality (245 vs. 212 mOsm·kg^−1^) [16]. Taken together, this data suggests that solution osmolality has a minor role in mediating gastrointestinal comfort during exercise. Rather, it seems likely that carbohydrate-induced gastrointestinal distress is primarily mediated by malabsorption, which would presumably be increased with during-exercise ingestion of slow-absorbing carbohydrate. In line with this hypothesis, others have reported increased incidences of gastrointestinal distress when carbohydrate is ingested during exercise at rates exceeding absorption capacity [6,36]. It is worth noting that ratings of nausea were similarly elevated with Iso HMS and Low HMS despite substantial differences in during-exercise intake rates. Assuming that malabsorption was primarily responsible for elevations in feelings of nausea, one might expect that Iso HMS would result in more severe symptoms as a result of a presumably greater degree of malabsorption. It is possible that malabsorption-induced nausea does not respond sensitively to carbohydrate dose. Alternatively, the methods used to assess differences in gastrointestinal distress may have lacked sensitivity to determine subtle differences in symptom severity. More research is clearly warranted to further elucidate the mechanisms governing carbohydrate-induced gastrointestinal distress during exercise. 

Our finding that performance was enhanced with Iso HMS and G relative to Low HMS is in line with studies reporting dose-responsive effects of during-exercise carbohydrate ingestion on endurance performance [37,38]. However, prior investigations have only reported a dose-response effect for fast-absorbing carbohydrates (i.e., maltodextrin, glucose, and fructose) with the effect seemingly being mediated by carbohydrate oxidation efficiency. Specifically, performance is optimized when the maximal amount of carbohydrate is ingested than can feasibly be absorbed. Maltodextrin/fructose composites ingested at maximally-absorbable rates (90 g·h^−1^) maximize performance relative to the same dose of maltodextrin (or lower doses of maltodextrin/fructose) because it can be taken up via separate intestinal transporters permitting absorption of a greater total amount of carbohydrate relative to what is ingested for a given unit of time (e.g., g·min^−1^) [7]. While oxidation efficiency of HMS has not been measured, it would be expected to be relatively low based on its low glycemic index of 32 and studies reporting that exogenous oxidation rates of similarly slow-absorbing carbohydrates is roughly half that of glucose [27,39]. Thus, this previously-reported dose-response effect may not be a function of oxidation efficiency, but rather is solely a function of carbohydrate quantity. Indeed, while G outperformed Low HMS, Iso HMS and G performance was no different, despite likely different oxidation efficiencies. 

Nevertheless, our finding of a slow-absorbing carbohydrate dose-response for performance is uncertain in light of our mechanism analyses. Gastrointestinal distress had a clear negative effect on performance with Low HMS vs. G. In fact, the likely 5% performance impairment with Low HMS vs. G became an unclear 0.4% enhancement when adjustments were made for gastrointestinal distress. This finding would suggest that, independent of gastrointestinal distress, carbohydrate dose had no impact on performance. However, adjusting for gastrointestinal distress had no clear impact on the 4.4% improvement in performance with Iso HMS vs. Low HMS suggesting that higher doses of HMS relative to lower doses improve performance even independent of gastrointestinal distress. For an explanation for these seemingly conflicting findings, it is likely that the similar levels of gastrointestinal distress between Iso and Low HMS trials confounded any adjustment for this covariate. More research is warranted to determine the extent to which performance responds (if at all) to HMS dose and how it is impacted by gastrointestinal distress.

Other interesting findings of the present study include an attenuated heart rate during steady state exercise and attenuated blood glucose concentrations following sprint 10 with HMS vs. G. The elevations in heart rate with G may have been due to the well-documented stimulatory effect of oral glucose on motivation and pleasure centers in the brain augmenting motor output [40]. Indeed, in a recent, (but yet to be published), study examining the impact of mouth rinsing with glucose on fatigued cyclists (following ~2.5 h of cycling), heart rate was elevated during subsequent steady-state exercise (50% W_max_) following the glucose, but not placebo, rinse (Dr. Nicholas Luden, personal communication [41]). Late-exercise differences in blood glucose were likely the result of a mismatch between muscle uptake of blood glucose, which was likely high late in exercise, and exogenous blood glucose delivery, which would presumably be slower/reduced with HMS relative to G. 

## 5. Conclusions

Findings from the present study suggest that ingesting HMS at currently-recommended rates before and during exercise maintains euglycemia, increases fat oxidation, and reduces carbohydrate oxidation during exercise in trained male cyclists. However, HMS has no impact on high-intensity cycling performance compared to fast-absorbing carbohydrate and is associated with gastrointestinal distress. Reducing the intake rate of HMS during exercise does not attenuate the risk of gastrointestinal distress, and it impairs performance. As such, the value of HMS as a during-exercise supplement seems limited. Future research should examine alternative dosing strategies designed to enhance gastrointestinal tolerance and examine the influence of gut trainability for HMS supplements. Additionally, continued research on potential applications of HMS as a pre-exercise supplement should be explored. 

## Figures and Tables

**Table 1:** Means and pairwise comparisons for oxygen consumption, total carbohydrate oxidation, and fat oxidation during steady state exercise.

Mean			VO_2_ (L∙min^−1^)	CHO Oxidation (g∙min^−1^)	Fat Oxidation (g∙min^−1^)
**Rest**	G		0.33 (23.5)	0.22 (58.6)	0.07 (82.1)
	Iso HMS		0.33 (7.9)	0.11 (89.6)	0.12 (23.8)
	Low HMS		0.32 (14.7)	0.09 (144.5)	0.12 (49.6)
**Exercise**	G		2.51 (9.0)	1.95 (8.7)	0.44 (40.6)
	Iso HMS		2.46 (9.3)	1.58 (21.0)	0.58 (34.7)
	Low HMS		2.48 (10.0)	1.60 (23.3)	0.56 (38.4)
Relative Difference (%); ±90% Confidence Interval *					
**Rest**	Low HMS–G	Mean effect	−2.2; ±11.2	−144.7; ±162.7	38.2; ±17.1
		Inference	unclear	very likely large	very likely moderate
	Iso HMS–G	Mean effect	−0.9; ±10.9	−48.9; ±21.4	64.0; ±62.2
		Inference	unclear	very likely moderate	very likely moderate
	Iso HMS–Low HMS	Mean effect	1.3; ±7.6	48.1; ±127.7	1.5; ±27.2
		Inference	unclear	unclear	unclear
**Exercise**	Low HMS–G	Mean effect	−1.2; ±3.0	−22.1; ±12.9	20.9; ±16.1
		Inference	possibly trivial	very likely very large	likely moderate
	Iso HMS–G	Mean effect	−2.1; ±2.3	−19.2; ±7.6	31.6; ±20.1
		Inference	possibly small	most likely very large	very likely moderate
	Iso HMS–Low HMS	Mean effect	−1.0; ±1.9	−1.4; ±12.8	4.1; ±22.1
		Inference	likely trivial	unclear	unclear

Note: Data for mean responses is presented as mean (CV). Exercise data was collected during 0–60 min of exercise. G, a glucose and sucrose-based supplement; Low HMS, low dose of hydrothermally-modified starch; Iso HMS, an isocaloric dose (relative to G) of hydrothermally-modified starch; CHO, carbohydrate. * Determination of inferences and effect sizes is described in the methods section.

**Table 2:** Pairwise comparisons for perceptual responses during repeated sprints.

Treatment Comparisons		Perceptual Response Difference (Scale Units)					
		Nausea	Abdominal Cramp	Fullness	Effort	Tiredness	Leg Strength
Low HMS–G	Mean effect	17.9; ±14.1	5.0; ±6.1	1.9; ±8.0	1.5; ±3.5	1.4; ±5.6	−2.4; ±7.5
	Inference*	likely moderate	likely moderate	unclear	likely trivial	unclear	unclear
Iso HMS–G	Mean effect	17.2; ±18.2	2.1; ±7.1	5.9; ±11.8	−2.3; ±4.0	4.9; ±5.5	−4.8; ±5.6
	Inference	likely moderate	unclear	unclear	likely trivial	possibly small	possibly small
Iso HMS–Low HMS	Mean effect	−0.7; ±16.9	−2.8; ±4.1	4.0; ±7.8	−3.8; ±6.3	3.6; ±4.5	−2.4; ±6.6
	Inference	unclear	possibly small	possibly trivial	possibly trivial	possibly small	unclear

Note: Data is presented as scale unit differences between treatments ±90% confidence interval. G, a glucose and sucrose-based supplement; Low HMS, low dose of hydrothermally-modified starch; Iso HMS, an isocaloric dose (relative to G) of hydrothermally-modified starch; * determination of inferences and effect sizes is described in the methods section.

**Table 3:** Effect of gastrointestinal distress on mean sprint power.

	Relative Difference (%) in Mean Sprint Power		
	Low HMS–G	Iso HMS–G	Iso HMS–Low HMS
Unadjusted mean sprint power	−5.0; ±5.3	−0.6; ±3.0	4.4; ±3.2
	likely small	likely trivial	likely small
Effect of gastrointestinal distress	−5.5; ±2.2	−1.4; ±1.4	−0.3; ±0.2
	very likely small **	unclear	Unclear **
Effect independent of gastrointestinal distress	0.4; ±3.5	0.8; ±3.1	4.7; ±2.7
	Unclear **	likely trivial	likely small
Effect of Individual Symptoms			
Effect of nausea	−5.2; ±2.7	−1.4; ±1.6	0.1; ±0.1
	Unclear **	likely trivial **	Unclear **
Effect independent of nausea	0.1; ±4.2	0.9; ±3.2	4.4; ±2.7
	Unclear **	likely trivial **	likely small
Effect of abdominal cramp	−2.9; ±2.0 **	−0.3; ±0.6	0.4; ±1.4
	unclear	most likely trivial **	Unclear **
Effect independent of abdominal cramp	−2.0; ±4.4 **	−0.2; ±3.1	4.0; ±3.7
	possibly trivial	Unclear **	possibly small **

Note: Data is presented as relative differences between treatments ±90% confidence interval. G, a glucose and sucrose-based supplement; Low HMS, low dose of hydrothermally-modified starch; Iso HMS, an isocaloric dose (relative to G) of hydrothermally-modified starch; * gastrointestinal distress refers only to effects of nausea and abdominal cramp because ratings of fullness did not correlate with changes in performance; ** indicates a change in effect magnitude and/or inference mediated by the covariate.

## Abbreviations

The following abbreviations are used in this manuscript:

CV	coefficient of variation
ES	effect size
FFA	free fatty acids
G	glucose and sucrose-based carbohydrate supplement
HMS	hydrothermally modified starch
VO_2_	oxygen consumption
VO_2max_	maximal oxygen consumption
W_max_	maximal cycling power
